# A Review on the Challenges in Indian Genomics Research for Variant Identification and Interpretation

**DOI:** 10.3389/fgene.2020.00753

**Published:** 2020-07-22

**Authors:** Sandhya Kiran Pemmasani, Rasika Raman, Rajkishore Mohapatra, Mathukumalli Vidyasagar, Anuradha Acharya

**Affiliations:** Research and Development Division, Mapmygenome India Limited, Hyderabad, India

**Keywords:** clinical genomics, variant classification, Indian genomics research, Indian genomic databases, machine learning

## Abstract

Today, genomic data holds great potential to improve healthcare strategies across various dimensions – be it disease prevention, enhanced diagnosis, or optimized treatment. The biggest hurdle faced by the medical and research community in India is the lack of genotype-phenotype correlations for Indians at a population-wide and an individual level. This leads to inefficient translation of genomic information during clinical decision making. Population-wide sequencing projects for Indian genomes help overcome hurdles and enable us to unearth and validate the genetic markers for different health conditions. Machine learning algorithms are essential to analyze huge amounts of genotype data in synergy with gene expression, demographic, clinical, and pathological data. Predictive models developed through these algorithms help in classifying the individuals into different risk groups, so that preventive measures and personalized therapies can be designed. They also help in identifying the impact of each genetic marker with the associated condition, from a clinical perspective. In India, genome sequencing technologies have now become more accessible to the general population. However, information on variants associated with several major diseases is not available in publicly-accessible databases. Creating a centralized database of variants facilitates early detection and mitigation of health risks in individuals. In this article, we discuss the challenges faced by genetic researchers and genomic testing facilities in India, in terms of dearth of public databases, people with knowledge on machine learning algorithms, computational resources and awareness in the medical community in interpreting genetic variants. Potential solutions to enhance genomic research in India, are also discussed.

## Introduction

Dynamic migration history, ethnic and genetic diversity and a high degree of consanguinity contribute to the complex and heterogeneous nature of the Indian population. There are many known genetic diseases affecting different population subgroups and insufficient scientific resources to diagnose and treat them (Aggarwal and Phadke, 2015; GUaRDIAN Consortium et al., 2019). Large-scale genetic studies in Indian patients are required to study disease-causing mutations and to develop personalized treatment methods. Another important aspect is accurate analysis and interpretation of genetic data. While tried and tested statistical methods work fairly well for biomarker discovery, advanced solutions like machine learning algorithms bring a promise of genomics driven clinical solutions. In this article, we discuss the current scope of Indian genomics in healthcare, challenges in scientific resources and data analysis, and solutions to enhance genomic medicine in India.

## Current State of Genetic Testing

Genetic testing in India has evolved in leaps and bounds in the past decade. Currently, there exist DNA-based tests that address multiple concerns in healthcare, from disease prevention to molecular diagnosis (Kar and Sivamani, 2016). In the case of preventive healthcare, genetic tests estimate the lifetime risk of disease, predisposition to biological traits and health parameters (Mohan et al., 2011). They also analyze a person’s response to drugs in terms of efficacy and risk for adverse reactions. These tests are primarily used as screening tools for establishing an effective strategy to reduce disease risk, delay, or avoid symptoms and manage existing conditions. Diagnostic genetic tests, on the other hand, help in identification of the molecular cause of the disease. These tests are used to confirm known or suspected diagnosis, carrier status determination, identification of at-risk genetic relatives, optimize treatments, and clinical decisions (Gupta et al., 2017; Aravind et al., 2019; Uttarilli et al., 2019). There are different types of diagnostic genetic tests currently available in India such as single-gene and multigene testing, exome, and genome sequencing, carrier and newborn screening (Puri et al., 2017; Singh et al., 2018). Other types of tests include those which assess reproductive risk, such as prenatal testing and preimplantation genetic diagnosis (Dada et al., 2008).

### Challenges and Limitations

Understanding the need of the patient is the key for determining the right genetic test. The biggest hurdles faced by clinicians are genetic data interpretation, finding genetic links for complex conditions, and lack of actionable genetic information. In certain cases of complex conditions, such as cancer, an array of genetic tests might be ordered to determine the genetic cause (Prabhash et al., 2019). However, no findings may come to light, thus posing a challenge for the patient and the clinician. The accuracy and precision of genetic tests lies in the translation of genetic findings into clinical outcomes. In the absence of information on genotype-phenotype correlations, genetic test results might be inconclusive.

## Genetic Data Interpretation for Indian Patients

Genetic diagnosis via clinical sequencing (e.g., genome-, exome-, single-, or multi-gene) is the front-line test recommended for many inherited diseases (Verma et al., 2018; Ganapathy et al., 2019). Establishing the genetic cause of disease is vital for patient care and treatment, and hence clinical findings must be reported with high precision and accuracy (Singh et al., 2016). All clinical reporting protocols are required to adhere to standards set by American College of Medical Genetics (ACMG), for proper classification of variants and subsequent disclosure to patient/clinician (Richards et al., 2015). As per ACMG guidelines, in order to differentiate benign and pathogenic variants, a detailed study of the variant’s clinical significance is required. This includes multiple criteria such as variant frequency, location in or near the gene, mechanism of said gene, effect of variant on protein domain or function, hotspot, or nearby mutations if any, etc. Apart from these, evidence of the variant having caused the disease in patients with similar clinical phenotype is essential to establish pathogenicity.

### Challenges and Limitations

Currently, there is a dearth of publicly available resources that provide an extensive list of clinically significant variants in Indian patients, for several genetic diseases. In the absence of published literature for a particular variant, which clearly is not benign, the variant gets classified as a variant of uncertain significance (VOUS). Interpreting VOUS is often challenging as they are not actionable, yet hold potential for establishing pathogenicity. For accurate classification and high-precision reporting of genetic variants, it is vital that geneticists and scientists have access to information on the complete spectrum of variants and mutations in Indian patients (Rajasimha et al., 2014; Genomics and other Omics tools for Enabling Medical Decision, 2019). Only the most relevant mutations are listed in databases like OMIM, which use selection criteria such as frequency, phenotype, significance, disease mechanism, and inheritance, etc.

### Case Study: Retinitis Pigmentosa

Retinitis Pigmentosa (RP) represents a very large group of eye disorders, with different clinical features, and symptoms. RP can be inherited in an autosomal dominant, autosomal recessive, or *X*-linked manner. Genetic diagnosis of RP helps in establishing genetic cause of disease, screening in at-risk family members and clinical management. But there are limited studies which report population specific mutations in Indian RP patients (Table 1).

**TABLE 1:** Information on summary of mutation studies in Indian RP patients.

**Number of patients**	**Significant findings**	**Gene**	**Method**	**Variant information available**	**References**
14 Families (autosomal recessive RP) and 100 cases (sporadic RP)	12 Novel mutations	EYS	Whole exome sequencing of proband followed by targeted analysis in family members	Yes	Di et al., 2016
1 Family and 100 cases (sporadic)	4 Novel mutations	CRB1		Yes	Yang et al., 2016
1 Family (autosomal recessive RP)	1 Novel mutation	MERTK		Yes	Bhatia et al., 2018b
1 Family (non-syndromic autosomal dominant RP)	1 Novel mutation	PRPF31	Targeted sequencing	Yes	Bhatia et al., 2018a
1 Family (autosomal recessive RP)	1 Novel mutation	MERTK	Microarray	Yes	Srilekha et al., 2015
2 Families (autosomal recessive RP) and 100 cases (sporadic RP)	1 Novel mutation	FAM161A	Whole exome sequencing	Yes	Zhou et al., 2015
2 Families (autosomal recessive RP)	1 Novel mutation	NR2E3	Microarray	Yes	Kannabiran et al., 2012
101 Cases (48 isolated cases and 53 autosomal dominant RP cases)	2 Novel mutations	PRPF31	Capillary sequencing	Yes	Gandra et al., 2008

Let us examine a case study of a 42 year old male reported with personal medical history of RP, who had been diagnosed at 16 years of age. His sister and two paternal cousins were also affected with RP. Married to a non-consanguineous partner, there were no clinical conditions in his children – 16 year old son and 12 year old daughter. Exome sequencing was done at Mapmygenome (Mapmygenome, 2020) to identify disease causing gene mutations associated with RP. Exome analysis revealed a heterozygous missense variant in exon 4 of the *NR2E3* gene. The observed variant is not reported as a variant in the normal samples of 1000 Genomes database and has a minor allele frequency of 0.018% in the gnomAD database. The variant is conserved across the species and *in silico* prediction by Mutation taster was found to be damaging. Another missense variant in this gene, Pro152Ser, has previously been reported with “retinitis pigmentosa 37,” and “retinitis pigmentosa (recessive)” in clinvar (Clinvar, 2019) as VOUS. In Indian Genetic Disease Database (IGDD), which is the first patient based genetic disease database of India (Pradhan et al., 2011) only seven genes have been mapped for RP. No variant was reported from the *NRE23* gene in IGDD. There are no other databases which have mutation information from Indian RP patients. Since there is no functional or published study of *NR2E3* mutations in Indian RP patients, Mapmygenome had classified the variant in exon 4 of the gene as a VOUS. There is insufficient evidence to establish this variant’s pathogenicity.

## Available Resources With Genotype-Phenotype Associations in Indian Population

Databases which host information on gene variants and associated diseases help genome analysts to make clinically significant and medically actionable inferences. However, most of the publicly available Indian databases are incomplete. This can be attributed to legal, ethical, financial, or administrative procedures due to which a lot of key parameters do not get recorded. Some of the Indian-specific databases, along with their scope and utility, have been discussed below.

### Index-dB

A database of exonic variants from normal individuals of Indian sub-continent (Ahmed et al., 2019). It is a user-friendly database with a querying feature and a browser to search for the variants. But the current version is based only on 109 individuals and is still under development.

### TMC-SNPdB

Contains variants generated from exome data of normal samples derived from tongue, gall bladder, and cervical cancer patients of Indian origin (Upadhyay et al., 2016). The major limitation of the database is not only the sample size of 62, but also the way the variants were processed. The COSMIC database was used to filter out somatic variants, because of which some novel Indian variants might have got filtered out.

### SAGE

A repository of genetic variants derived through an integration of six datasets comprising 1213 South Asian genomes and exomes (Judith et al., 2018). It contains more than 154 million variants, out of which 69 million are novel variants. Though this a comprehensive database of South Asians, it should be enriched with region or ethnicity specific datasets within South Asia.

### Indian Genetic Disease Database (IGDD)

A curated database of variants associated with diseases prevalent in Indian population (Pradhan et al., 2011). Diseases were categorized into different therapeutic areas. The current version of the database covers 104 diseases with a total of ∼3500 patients. Further enrichment is required to cover more diseases in the population.

### Indian Genome Variation Database (IGVdB)

This was started as a consortium activity in 2003, with the goal to create a variation database of Indian population (Indian Genome Variation Consortium, 2005; Narang et al., 2010). However, this database does not contain disease-variant associations, which are helpful in interpreting the data obtained from genetic tests.

### GWAS Central – India

A genotype-phenotype association database with summary level findings from genetic association studies (Indian GWAS, 2010). Lack of regular updates and absence of extensive data points for genetic diagnosis, make this database a less effective tool for clinicians, or bioinformaticians, thereby limiting its clinical utility.

### Indian SNP Data

Contains genotype data of 871,771 SNPs, obtained from 15 Dravidian trios, and 13 Indo-European trios (Indian SNP, 2020). Browser and query features are not available for this database. Files can be downloaded for academic and research purposes only. Although it was initially developed as a reference panel for Indians, it has limited data and the work is still in progress.

### Genotype/Phenotype DB

This database contains genotype and phenotype data of Indian population along with their demographic details (CCMB, 2020). Browser and query features are not available for this database. Commercial organizations are strictly prohibited from using the data.

### Indigen Project

This is an initiative from Council of Scientific and Industrial Research (CSIR) for whole genome sequencing of 1000 Indian genomes, across diverse ethnic groups, with the goal to enable clinical applications in rare genetic diseases. This is an initiative which is yet to see fruition and is yet to be publicly available for the scientific community (IndiGen, 2020).

The above databases have not been presented in a way that allows the user to understand the pathogenicity of variants. Genomics companies like Mapmygenome (Mapmygenome, 2020), do not have access to most of such databases. A centralized database curated from Indian patients, for different diseases, would help in precise reporting and clinical decision making.

Publicly available data and results generated from genome wide association studies (GWAS) can also be utilized in interpreting the variants and in identifying new variants. There are case-control association studies done on Indian population, for majorly occurring diseases – Type 2 Diabetes, cardiovascular diseases and cancers (Chauhan et al., 2010; Nagrani et al., 2017; Bellary et al., 2019). Polygenic Risk Scores (PRS) developed from GWAS act as prognostic indicators in preventive healthcare. However, reliability of the results depends on the algorithm used and the data available.

## Machine Learning (ML) Algorithms in Indian Genomics

With the availability of diverse data types – gene expression, SNP genotypes, demographics, heath history, laboratory findings, and images etc. – machine learning algorithms have become the obvious choice for accurate prediction of disease risk and personalized treatment. They can learn patterns underlying complex data and build models that can be used for prediction purposes. Numerous machine learning methods, such as support vector machines, random forests, and Bayesian networks, are being used successfully in genomics research and applications (Libbrecht and Noble, 2015; Xu and Jackson, 2019). Now, deep learning algorithms, a subcategory of machine learning, have emerged as the most successful algorithms for combining clinical data with genomics (Ching et al., 2018; Zou et al., 2019). They use artificial neural networks to progressively extract novel features from input data and learn from the features (Eraslan et al., 2019).

Deep learning and machine learning algorithms, which come under the umbrella term Artificial Intelligence (AI), are being used in clinical practice through numerous commercial applications involving clinical and genomics data. A well known personal genomics company, 23andme (2020) uses machine learning algorithms in disease risk prediction. IBM’s Watson for Oncology (IBM Watson for Oncology, 2020) helps clinicians in identifying most appropriate treatment options based on information collated from medical records, medical journals, genomic journals, and relevant guidelines. Many startups are increasingly using the combination of machine learning algorithms and genomics in creating tools and processes that enhance the healthcare systems. For example, Freenome (2019), Benevolent AI (2020), Cambridge Cancer Genomics (2020), and DeepGenomics (2020) use AI in predicting disease risk, response to therapy and in developing personalized treatment regimens. In India, very few organizations use machine learning algorithms in clinical genomics, with the reasons being lack of awareness and lack of expertise in research and application of AI. Some of the Indian pharmaceutical and genomic organizations that are using AI include Innoplexus (2019), Lantern Pharma (2019), Manipal Group of Hospitals (2019), TCS Innovation Labs (2019), BioXcel Therapeutics Inc. (2020), Mapmygenome (2020), OncoStem (2020), and PierianDx (2020).

### Challenges and Limitations

Main technical challenges in the application of ML algorithms are data curation and data pre-processing (Ngiam and Khor, 2019). Different hospitals and laboratories adopt different terminologies to record a disease or a health condition and use different reference ranges. In India, Electronic Health Records Standards were released by the Ministry of Health and Family Welfare in 2016. But sharing of data between the hospitals through a common platform is still a work in progress.

Data sets used in training the machine learning algorithms should clearly represent the target data for which risk predictions are made. For example, genetic algorithms trained on data from North Indians might make less accurate predictions when applied on South Indians. Comprehensive and robust clinical data sets that represent the ethnic differences among the people of India are still unavailable. To facilitate sharing of biological data across various research organizations in India, especially high-throughput data generated by sequencing and microarrays, and to create National Biological Data Centre, Ministry of Science and Technology has released zero draft on Biological data storage, access and sharing policy of India in July 2019 (Department of Science and Technology, 2020). But it is still in its nascent stage. A standard procedure for normalizing the raw data must be developed to maintain uniformity across the research groups.

Lack of understanding among clinicians and patients about the machine learning algorithms and their predictions make them considered as black box algorithms (Vayena et al., 2018). Data scientists should explain the general logic behind the algorithm-based decisions. Doctors and patients should understand the risk associated with such decisions. Clear communication between data scientists, doctors and patients is required to maintain ethical standards in clinical applications.

### Solutions to Overcome the Challenges

NITI Aayog, a policy think tank of the government of India, made several recommendations to address the challenges and to harness the power of AI in India (National Strategy for AI, 2018). They include – establishing Centres of Research Excellence (COREs), increasing R&D resources, supporting Ph.D. researchers, establishing common supercomputing facilities, and creating an ecosystem for development and application of AI. Encouraging institute-industry partnerships, creating investment funds for AI startups and reskilling the existing workforce have also been discussed in detail. Other research agencies like Itihaasa (2018) made similar recommendations.

Institutional review boards, ethical review committees and scientific societies should come up with best practices for application of ML in clinical genomics. Government should start a regulatory body in lines similar to the United States Food and Drug Administration (FDA) to enforce best practices. Data sets used in training the algorithms, variables considered in building the models and accuracy of the predictions should be scrutinized. Updating the models by retraining the algorithms and checking the efficiency of the models should be done in coordination with the clinicians. The Government of India should take initiatives to train clinicians in understanding machine learning algorithms. Certification programs run through premier institutes would encourage the people to take up such courses.

## Ethical and Legal Concerns in Data Sharing

Genomic data is sensitive in nature and public sharing of such data brings a fair share of ethical and legal concerns with it. Given the increasing number of direct-to-consumer tests that are available, there is a need to streamline certain processes. The collection, storage and usage of genetic data must enable meaningful outcomes for personalized medicine. Data security and privacy remains one of the major concerns reported by users. The “Personal Genomes: Accessing, Sharing and Interpretation” conference held in the United Kingdom, in April 2019 (Genetics Society, 2019) addressed several conundrums which hinder sharing of genetic and medical data, for the creation and maintenance of genomic databases. There is also a growing segment of users who are open to sharing their de-identified data (Kim et al., 2015; Rubin and Glusman, 2019). They share their data for getting updates on their health reports, for providing social good or for financial compensation (Hendricks-Sturrup and Lu, 2020). In India, with the release of Personal Data Protection Bill 2019 (The Personal Data Protection Bill, 2019) certain principles were laid down on collection and usage of personal data. Informed consent, data minimization and storing a copy of data within India are some of the essential requirements under the bill.

The benefits of sharing genomic data in the scientific community are far too many to ignore. Collaborative efforts between sequencing facilities, data scientists, clinics, and healthcare providers must be directed toward building a healthy ecosystem for data sharing. De-identification of the genetic information as well as medical records is essential. Wright et al. (2019) proposes a system wherein genetic variant details and their associated conditions can be shared in online databases, without requiring explicit consent from patients. However, detailed clinical information and case study at a deeper level will require consent from the doctors and their patients. For the Indian scenario, a robust system for data sharing is required. This system must be regulated by measures which protect the patients’ interests as well. Policy makers and leaders must come together to develop a framework that allows more variant databases to become publicly accessible, without breach of privacy.

## Role of Clinicians in Indian Genomics

Clinicians play a very important role in facilitating genomics-driven healthcare. From the time a patient visits the clinic to the time of treatment, there are several stages that require the clinician to relay information related to testing procedures and their possible outcomes. The clinician holds a key responsibility of comprehending the implications of genetic findings and making the necessary correlations for treatment and management. Hence, it is imperative that the clinician is well versed with different genetic mechanisms, inheritance, gene-gene, and gene-environment interaction mechanisms, variants and their pathogenicity. In the clinic, staff must be trained to perform timely reviews of clinical and family history and identify cases which warrant genetic testing. For the current generation of clinicians, training on genetic diseases, testing methodologies, clinical variant interpretation and application in medicine, must be included as part of their continuing education. Policy makers such as Medical Council of India and Board of Education play an important role in training clinicians on utilizing genomics in their practice (Scheuner et al., 2008; Aggarwal and Phadke, 2015).

## Conclusion

Given the broad spectrum of genetic diseases and their burden on the Indian population, it is essential for genomic researchers to tap Indian genetic data for disease prevention, timely diagnosis, and treatment. Studies show that there are novel mutations in Indian patients, for different phenotypes. Hence, genome analysts need to refer to Indian-specific databases for meaningful translation of genomics data into clinical reporting. Current challenges can be met by united efforts from government health agencies and genetic research institutes by executing large scale sequencing projects, accompanied by detailed documentation on patients’ clinical features and family history. Obtaining informed consent from the patients must be mandatory, to protect their interests including concerns about data privacy and safety. The patients must be educated about protocols such as de-identification, data security and research objectives.

Novel variants must be made available in a centralized database for analysts to refer to, and draw inferences from. Such a database would vastly improve the diagnostic accuracy of genetic diseases. Indian genomics will also greatly benefit by the development of machine learning algorithms for analyzing health trends in the Indian population. Additionally, clinicians from all walks of medicine must be equipped with technical knowledge on medical genetics and its clinical application, for enhanced patient care.

## Author Contributions

SP and RR have contributed conception and design of the study. SP, RR, and RM wrote sections of the manuscript. MV and AA have supervised and reviewed the manuscript. All authors contributed to the article and approved the submitted version.

## Conflict of Interest

SP, RR, RM, and AA are employed by the company Mapmygenome India Limited. MV is a member of Scientific Advisory Board at Mapmygenome India Limited.
